# Left ventricular assist device driveline infections in three contemporary devices

**DOI:** 10.1111/aor.13843

**Published:** 2020-11-28

**Authors:** Thomas Schlöglhofer, Peter Michalovics, Julia Riebandt, Philipp Angleitner, Martin Stoiber, Günther Laufer, Heinrich Schima, Dominik Wiedemann, Daniel Zimpfer, Francesco Moscato

**Affiliations:** ^1^ Center for Medical Physics and Biomedical Engineering Medical University of Vienna Vienna Austria; ^2^ Division of Cardiac Surgery Medical University of Vienna Vienna Austria; ^3^ Ludwig‐Boltzmann‐Institute for Cardiovascular Research Vienna Austria

**Keywords:** driveline infection, mechanical circulatory support, readmission, risk factors, ventricular assist device

## Abstract

Driveline infections (DLI) are common adverse events in left ventricular assist devices (LVADs), leading to severe complications and readmissions. The study aims to characterize risk factors for DLI readmission 2 years postimplant. This single‐center study included 183 LVAD patients (43 HeartMate II [HMII], 29 HeartMate 3 [HM3], 111 HVAD) following hospital discharge between 2013 and 2017. Demographics, clinical parameters, and outcomes were retrospectively analyzed and 12.6% of patients were readmitted for DLI, 14.8% experienced DLI but were treated in the outpatient setting, and 72.7% had no DLI. Mean C‐reactive protein (CRP), leukocytes and fibrinogen were higher in patients with DLI readmission (*P* < .02) than in outpatient DLI and patients without DLI, as early as 60 days before readmission. Freedom from DLI readmission was comparable for HMII and HVAD (98% vs. 87%; HR, 4.52; 95% CI, 0.58‐35.02; *P* = .15) but significantly lower for HM3 (72%; HR, 10.82; 95% CI, 1.26‐92.68; *P* = .03). DLI (HR, 1.001; 95% CI, 0.999‐1.002; *P* = .16) or device type had no effect on mortality. DLI readmission remains a serious problem following LVAD implantation, where CRP, leukocytes, and fibrinogen might serve as risk factors already 60 days before. HM3 patients had a higher risk for DLI readmissions compared to HVAD or HMII, possibly because of device‐specific driveline differences.

## Graphical abstract

Graphical presentation of the methods, results and implications of this study.

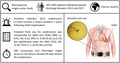

## 1 INTRODUCTION

Ventricular assist devices (VADs) are an established therapeutic option^1^ for patients with end‐stage heart failure and provide circulatory support until myocardial recovery, heart transplantation or as destination therapy. Over the last decade, mechanical circulatory support has further improved patient survival and quality of life due to improvements in the design and durability of the devices.^2^ However, infection remains a major adverse event and a relevant cause of morbidity and mortality in VAD recipients^2^ with considerable best practices variation regarding infection prevention and management and driveline exit site care.^3^, ^4^ Infection occurs in up to 60% of VAD patients^4^ and is the most frequent adverse event during the first 3 months and the most common adverse event thereafter.^2^ Three categories of infection were defined for VAD patients: VAD‐specific infections, VAD‐related infections, and non‐VAD infections.^5^ A VAD‐specific infection may involve the pump, cannula, pocket or driveline. Pump‐related percutaneous driveline infection (DLI) as the primary cause of readmission accounts for 13% of readmissions.^5^ DLI is mostly caused by biofilm‐producing bacteria and can lead to deep infections and sepsis if untreated, which is potentially lethal.^7^ Therefore, the analysis of risk factors as well as the early detection of DLI is crucial to optimize the results. The study aims to characterize the incidence of DLI, evaluate associations of DLI‐related readmissions with clinical outcomes and the pathogenic profile of DLI, and identify demographic or clinical parameters as risk factors and possible predictors for DLI readmission.

## 2 PATIENTS AND METHODS

### 2.1 Study population

This retrospective, single‐center study included 183 patients with left ventricular assist device (LVAD) support after initial discharge from the hospital between January 2013 and July 2017. The study protocol was approved by the Institutional Review Board. The primary endpoint of interest in this study was freedom from DLI readmission 2 years following implantation. Secondary outcomes included risk factors for DLI readmission and all‐cause mortality during the follow‐up period. We extracted the baseline characteristics and laboratory parameters of patients with DLI related readmission (RDLI), without readmission but DLI treated in the outpatient setting (NRDLI) and without DLI (NoDLI). DLI treatment strategies of the RDLI and NRDLI cohorts as well as the microbiological profile of DLIs were also evaluated. To identify clinical parameters as potential DLI related readmission predictors, laboratory findings during follow‐up at 30, 60, and 90 days preceding readmission and on the day of readmission were assessed in the RDLI group. In the NRDLI group, laboratory parameters assessed during follow‐up at the time of DLI diagnosis and all available laboratory values of NoDLI patients assessed during regular outpatient follow‐up without signs of DLI (no positive swab) were analyzed (Table 1).

**TABLE 1:** Demographics and laboratory parameters during follow‐up

	DLI readmission (n = 23)	DLI no readmission (n = 27)	*P* value*	No DLI (n = 133^a^)	*P* value**
Age at implant, years	59.0 (14.0)	56.5 (14.3)	.77	58.0 (14.8)	.75
Gender, male	22 (81.5)	21 (91.3)	.43	156 (85.0)	.77
Weight, kg	83.0 (22.3)	81.3 (16.0)	.68	82.8 (23.8)	.29
BMI, kg/m^2^	28.3 (7.0)	26.1 (6.5)	.33	26.4 (6.6)	.12
Smoking history, n (%)	15 (65.2)	10 (37.0)	**.045**	39 (29.3)	**.001**
Diabetes, n (%)	10 (47.8)	8 (29.6)	.22	42 (31.6)	.15
Device, n (%)					
HVAD	16 (69.6)	16 (59.3)	.76	79 (59.4)	**.04**
HeartMate II	2 (8.7)	3 (11.1)		38 (28.6)	
HeartMate 3	5 (21.7)	8 (29.6)		16 (12.0)	
Strategy, n (%)					
Destination therapy	7 (29.6)	(27.2)	.36	38 (28.9)	.20
Bridge to transplant	4 (18.5)	10 (36.4)		48 (36.7)	
Bridge to candidacy	12 (51.9)	10 (36.4)		46 (33.6)	
Bridge to recovery	0 (0.0)%	0 (0.0)		1 (0.8)	
INTERMACS level, n (%)					
1	2 (8.0)	3 (11.2)	.39	39 (29.6)%	.07
2	3 (12.0)	4 (14.8)		21 (16.0)%	
3	9 (40.0)	10 (37.0)		35 (25.6)%	
4‐7	9 (40.0)	10 (37.0)		38 (28.8)%	
Albumin, g/L	37.0 (7.4)	42.9 (4.2)	**<.001**	42.1 (5.3)	**<.001**
CRP, mg/dL	2.93 (18.78)	0.79 (2.20)	**<.001**	0.62 (1.10)	**<.001**
Leukocytes, g/L	10.41 (7.83)	8.17 (4.31)	**.026**	7.39 (2.88)	**<.001**
INR	2.55 (1.12)	2.60 (0.85)	.557	2.70 (0.70)	.549
aPTT, s	50.2 (21.1)	46.0 (11.7)	.094	45.3 (9.5)	**.001**
TCT, s	27.0 (–)	37.0 (19.0)	.548	30.0 (9.0)	.533
Fibrinogen, mg/dL	592 (220)	387 (125)	**<.001**	432 (123)	**<.001**

Note

Data presented as n (%) or mean ± standard deviation for normally distributed data or as the median with the interquartile range for non‐normally distributed data. Bold values indicates *P*‐values <0.05.

Abbreviations: aPTT, activated partial thromboplastin time; BMI, body mass index; CRP, C‐Reactive Protein; DLI, Pump‐related percutaneous driveline infection; INR, International Normalized Ratio; INTERMACS, Interagency Registry for Mechanically Assisted Circulatory Support; s, seconds; TCT, Thrombin Clotting Time.

^a^ Based on n = 617 laboratory samples collected from the 133 no DLI patients during follow‐up without any symptoms of DLI.

* *P* value reflecting statistical differences between DLI Readmission and DLI no Readmission group.

** *P* value reflecting statistical differences between DLI Readmission and no DLI group.

### 2.2 Postoperative dressing procedure

Driveline exit site dressing procedure was performed according to ISHLT consensus^4^ including aseptic technique, gloves, mask, and cap based on the same standard operating procedure for all patients. The wound dressings were applied with a mild antiseptic Octenidin solution (Octenisept, Schülke & Mayr GmbH, Norderstedt, Germany) and a no sting barrier film (Cavilon, 3M, Minneapolis, MN, USA). The driveline exit site was covered with a protective dressing including film compress with slit (Askina Pad 5 × 5 cm, B. Braun Hospicare Ltd., Sligo, Ireland) and a semipermeable foil (IV3000 10 × 12 × m, Smith & Nephew Medical Ltd, Hull, UK). Once healed and with no drainage present, the frequency of dressing was two to three times per week. Drivelines were immobilized with a binder (SECUTAPE Nanoplast fixation, TechniMed AG, Rorschach, Switzerland) to minimize the driveline movement.

### 2.3 Definition of LVAD‐related infections and infection management

VAD‐specific infections can be divided into superficial driveline or deep infections.^6^ LVAD‐related percutaneous driveline infections were defined^7^ as those requiring antimicrobial therapy when there were clinical signs of infection such as pain, fever, drainage from the exit‐site, and/or leukocytosis. As proposed by the Sharp Memorial group^8^ or the DESTINE staging proposal,^9^ DLI classification of stages 1 to 5 may be useful for the discussion of treatment strategy. If DLI was suspected, a complete infectious workup including information on bacterial cultures and daily wound care with a sterile silver‐impregnated dressing (AQUACEL Ag, ConvaTec, Munich, Germany) was performed for early treatment (stage 1 or 2) in the NRDLI cohort without readmission. Empirical antimicrobial therapy with a focus on *Staphylococcus* was initiated until a specific pathogen was isolated from the bacterial culture, including a switch to targeted therapy depending on sensitivity. In stage 3, characterized by a copious amount of drainage and tenderness, some patients had to be hospitalized. All patients with stage 4 or 5 were readmitted (RDLI) and treated with targeted antibiotic therapy based on the bacterial culture at the driveline exit site. For RDLI patients, surgical debridement and the use of a vacuum‐assisted closure device was possible. Successful treatment of DLI has been defined as the absence of clinical signs of infection, including a negative swab analysis of the bacterial culture taken by the driveline exit site.

### 2.4 Statistical analysis

Descriptive statistics are presented as mean ± standard deviation for continuous variables and number (percentage) for categorical variables. Where continuous variables were non‐normally distributed, data are presented as median and interquartile range. Normal distribution was assessed by the Shapiro‐Wilk test. Pearson's *χ* ^2^ or Fisher’s exact test was used to assess for statistical significance of categorical variables, Student's *t*‐test or Mann‐Whitney *U* test for continuous variables and one‐way ANOVA or Kruskal‐Wallis tests were used to test between more than two continuous groups. Clinical outcomes were compared between cohorts using hazard ratios (HRs) estimated from a Cox proportional hazards model for all‐cause death with DLI as a time‐dependent covariate. Clinically relevant risk factors for mortality and for the development of a DLI were chosen as covariates for this model. Kaplan‐Meier survival analysis using Mantel‐Cox statistics was used to examine time to first DLI readmission. Patient follow‐up was censored when patients underwent heart transplantation, device explantation or expired. Statistical significance was set at *P* < .05. Statistical analysis was performed by SPSS for Windows Release 26.0.0 (SPSS Inc, Chicago, IL, USA) and MATLAB R2017b (The MathWorks Inc, Natick, MA, USA).

## 3 RESULTS

### 3.1 Patient characteristics

The study population consisted of 183 continuous flow LVAD patients following initial discharge. Three different devices were implanted in our cohort: n = 43 (23.5%) HeartMate II (HMII) (Abbott Inc, Chicago, IL, USA), n = 29 (15.8%) HeartMate 3 (HM3) (Abbott Inc) and n = 111 (60.7%) HVAD (Medtronic Inc, Minneapolis, MN, USA). Patients (32.8%) received a VAD as bridge to transplant, 27.9% as destination therapy, 35.5% as bridge to candidacy, and 0.5% as bridge to recovery. The median age of the patients was 58.0 (14.0) years, median body mass index (BMI) was 26.6 (6.6) kg/m^2^, and 14.8% were female.

At 24 months, 23 (12.6%) patients had a DLI related readmission (RDLI) and 160 (87.4%) were without any DLI related readmission. Of the patients without readmission, 27 (14.7% of the entire cohort) experienced DLI but were treated in the outpatient setting (NRDLI) and 133 (72.7% of the entire cohort) had no DLI (NoDLI). Baseline demographics and laboratory parameters assessed on the day of readmission (RDLI, n = 23), during follow‐up at the time of DLI diagnosis (NRDLI, n = 27) or during follow‐up without any symptoms of DLI (no DLI, n = 133) are presented in Table 1. No DLI data were obtained from n = 617 laboratory samples (average 4.6 samples per patient). Age, gender, BMI, diabetes, smoking history, implant strategy, and Interagency Registry for Mechanically Assisted Circulatory Support (INTERMACS) level were not found to be risk factors for the development of DLI related readmission (Table 2).

**TABLE 2:** Independent risk factors for DLI related readmission (multivariable Cox proportional hazard model)

Variables	Hazard ratio	Confidence interval (95%)	*P* value
Age at implant, years	1.020	0.954‐1.089	.566
Gender, female	2.024	0.453‐9.038	.356
BMI, kg/m^2^	0.928	0.743‐1.160	.512
Smoking history	1.410	0.494‐4.024	.520
Diabetes	1.583	0.560‐4.476	.386
Device			
HeartMate II	ref		
HVAD	4.522	0.584‐35.024	.149
HeartMate 3	10.824	1.264‐92.681	**.030**
Strategy			
Bridge to candidacy	ref		
Bridge to transplantation	0.669	0.190‐2.353	.531
Destination therapy	0.128	0.016‐1.025	.053
Bridge to recovery	–	–	–

Bold values indicates *P*‐values <0.05.

Abbreviations: BMI, body mass index; DLI, pump‐related percutaneous driveline infection; ref, reference.

Significantly more RDLI patients (65.2%) had a smoking history pre‐LVAD implantation compared to NRDLI (37%, *P* = .045) and NoDLI (29.3%, *P* = .001) patients. Serum albumin levels were lower in the RDLI cohort [37.0 (7.4) g/L] compared to patients with NRDLI [42.9 (4.2) g/L, *P* < .001] and NoDLI [42.1 (5.3) g/L, *P* < .001]. Median C‐reactive protein (CRP) was higher in RDLI than in NRDLI and NoDLI patients (2.93 vs. 0.79 and vs. 0.62 mg/dL, *P* < .001)—similar results were found for leukocytes (10.41 vs. 8.17 and vs. 7.39 g/L, *P* < .05) and fibrinogen (592 vs. 387 and vs. 432 mg/dL, *P* < .001)—see Table 1. RDLI patients had significantly higher activated partial thromboplastin time (aPTT) than NoDLI (*P* = .001) and a trend toward higher aPTT versus NoDLI patients (*P* = .094). As shown in Table 1, no differences in international normalized ratio (INR) and thrombin clotting time (TCT) were found between RDLI, NRDLI, and NoDLI.

### 3.2 Microbiological profiles and DLI treatment strategies

The microbiological profile of DLIs demonstrated the predominance of *Staphylococcus* and *Pseudomonas* species in our LVAD cohort. *Staphylococcus aureus* was the most common pathogen in RDLI (52.0%) and NRDLI (59.4%), the second most common species was *Pseudomonas aeruginosa* in RDLI (16.0%) and *Staphylococcus epidermis* in NRDLI patients (12.5%). A summary of all pathogens detected in RDLI and NRDLI patients can be found in the supplementary online data, Table S1.

The DLI treatment strategies differed statistically significantly (*P* < .001) between the RDLI and NRDLI cohorts (Table 3). 33.3% of NRDLI patients were treated only with bacteriostatic silver dressing change and 66.7% with targeted oral antibiotics. In the RDLI cohort, all patients initially received targeted oral antimicrobial therapy, which in 22.2% of cases led to successful treatment of DLI. However, 51.9% of RDLI patients required IV antibiotics, 3.7% surgical debridement, and 22.2% vacuum‐assisted closure therapy for successful DLI treatment. The success rates of DLI treatments were comparable in RDLI and NRDLI patients (92.6% vs. 100%, *P* = .49).

**TABLE 3:** DLI treatment strategies and outcomes of patients with DLI stratified by readmission due to infection

	DLI readmission (n = 23)	DLI no readmission (n = 27)	*P* value
Treatment strategy, n (%)			
Bacteriostatic silver dressing change	0 (0.0)	9 (33.3)	**<.001**
Oral targeted antibiotics	5 (22.2)	18 (66.7)	
IV targeted antibiotics	12 (51.9)	0 (0.0)	
Surgical debridement	1 (3.7)	0 (0.0)	
Vacuum assisted closure therapy	5 (22.2)	0 (0.0)	
Treatment outcomes, n (%)			
Successful DLI treatment	21 (92.6)	27 (100.0)	.49
Died due to DLI	2 (7.4)	0 (0.0)	
Pump replacement	0 (0.0)	0 (0.0)	

Note

Data presented as n (%). Bold values indicates *P*‐values <0.05.

Abbreviations: DLI, driveline infection; iv, intravenous.

### 3.3 Prediction of DLI readmission

Laboratory parameters as potential DLI‐related readmission predictors up to 90 days before readmission are summarized in Table 4. The CRP at DLI readmission was significantly higher than 60 and 90 days before, but lower (*P* < .001) than 30 days before readmission. Fibrinogen was found to be higher at DLI readmission 592 (220) mg/dL versus 30, 60, and 90 days before [500 (157), 451 (224) and 496 (151) mg/dL, *P* < .05]. In contrast, leukocytes were significantly higher during DLI readmission than 90 days before [10.41 (7.83) vs. 7.18 (3.83), *P* = .019] but rather similar 30 and 60 days before readmission [9.28 (3.72) and 8.34 (3.19), *P* > .078].

**TABLE 4:** Laboratory parameters 30 to 90 days before DLI readmission

	DLI Readmission (n = 23)	30 days before Readmission (n = 14)	*P* value*	60 days before Readmission (n = 15)	*P* value**	90 days before Readmission (n = 16)	*P* value***
CRP, mg/dL	2.93 (18.78)	3.30 (3.36)	**<.001**	1.33 (2.67)	**<.001**	2.41 (4.33)	**<.001**
Leukocytes, g/L	10.41 (7.83)	9.28 (3.72)	.084	8.34 (3.19)	.078	7.18 (3.83)	**.019**
INR	2.55 (1.12)	2.40 (1.35)	>.99	1.80 (1.35)	.35	2.40 (0.70)	.086
aPTT, s	50.15 (21.1)	45.7 (4.1)	.094	45.0 (5.6)	>.99	45.1 (8.2)	.385
TCT, s	27.0 (–)	–	–	–	–	–	–
Fibrinogen, mg/dL	592 (220)	500 (157)	**.046**	451 (224)	**<.001**	496 (151)	**<.001**

Note

Data presented as n (%) or mean ± standard deviation for normally distributed data or as the median with the interquartile range for non‐normally distributed data. Bold values indicates *P*‐values <0.05.

* *P* value indicating statistical differences between DLI Readmission and 30 days before readmission.

** *P* value indicating statistical differences between DLI Readmission and 60 days before readmission.

*** *P* value indicating statistical differences between DLI Readmission and 90 days before readmission.

### 3.4 DLI readmissions and effect on outcomes

**FIGURE 1:**
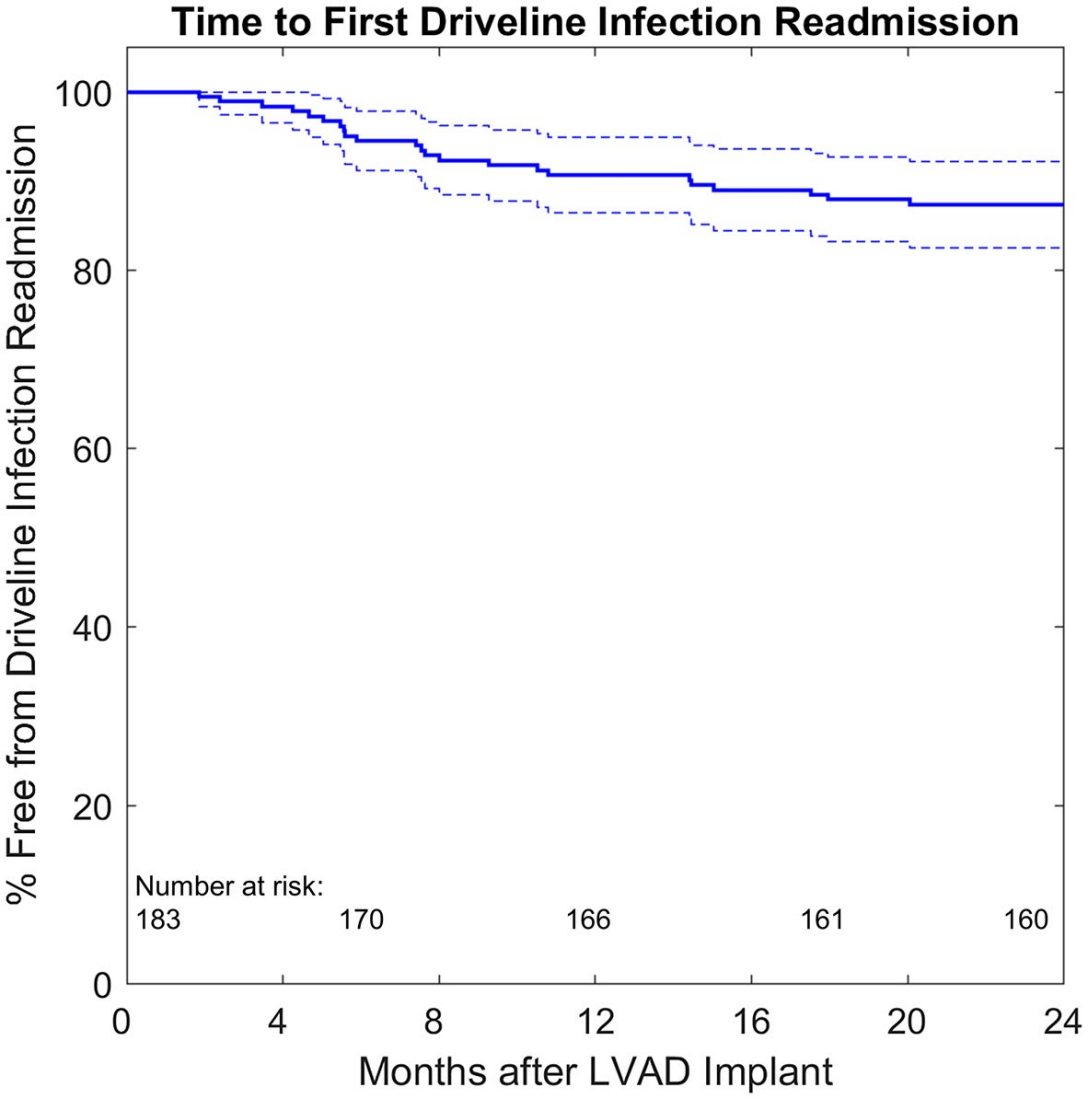
Freedom from the first driveline infection readmission. Dotted lines representing the 95% confidence intervals [Color figure can be viewed at wileyonlinelibrary.com]

Freedom from any pump‐related DLI readmission at 24 months was 87.4% (95% CI, 82.5%‐92.2%) (Figure 1). Long‐term survival during LVAD support (Table 5) was not affected by the occurrence of DLI (HR, 1.001; 95% CI, 0.999‐1.002; *P* = .16).

**TABLE 5:** Survival during LVAD support (multivariable Cox proportional hazard model with DLI as time‐dependent covariate)

Variables	Hazard ratio	Confidence interval (95%)	*P* value
Age at implant, years	1.062	1.006‐1.112	**.030**
Gender, female	0.306	0.072‐1.309	.110
BMI, kg/m^2^	1.056	0.886‐1.259	.540
Smoking history	1.629	0.698‐3.806	.259
Diabetes	1.457	0.732‐2.899	.284
DLI	1.001	0.999‐1.002	.155
Device			
HeartMate II	ref		
HVAD	0.734	0.302‐1.78	.494
HeartMate 3	0.387	0.076‐1.966	.252
Strategy			
Bridge to candidacy	ref		
Bridge to transplantation	0.107	0.030‐0.379	**.001**
Destination therapy	0.433	0.128‐1.130	.082
Bridge to recovery	–	–	–
INTERMACS level	0.996	0.714‐1.389	.982

Bold values indicates *P*‐values <0.05.

Abbreviations: BMI, body mass index; DLI, pump‐related percutaneous driveline infection; INTERMACS, Interagency Registry for Mechanically Assisted Circulatory Support; LVAD, left ventricular assist device; ref, reference.

**FIGURE 2:**
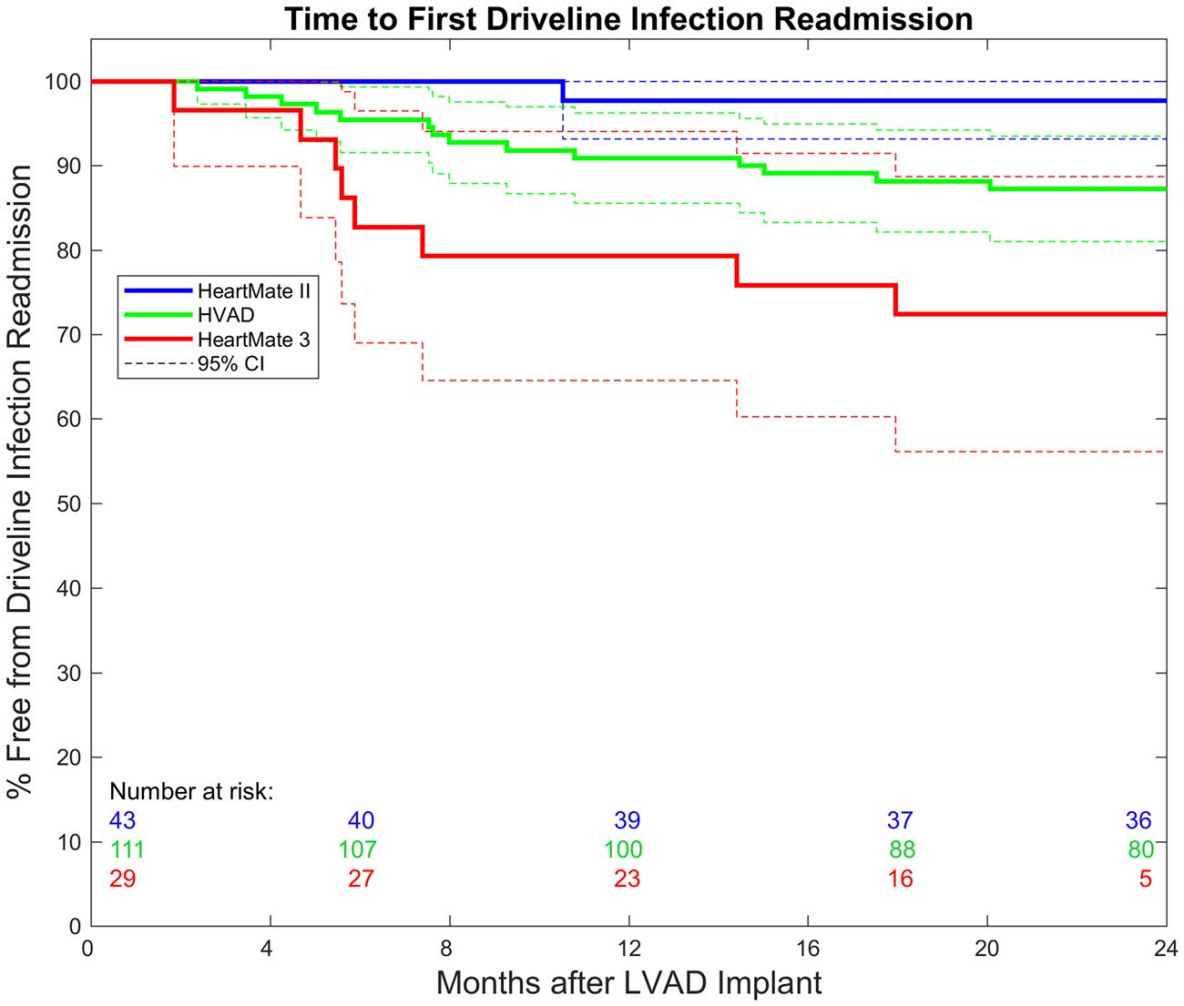
Freedom from first driveline infection readmission, stratified by device type. Dotted lines representing the 95% confidence intervals [Color figure can be viewed at wileyonlinelibrary.com]

Freedom from any DLI readmission was comparable for HMII and HVAD (98% vs. 87%; HR, 4.52; 95% CI, 0.58‐35.02; *P* = .15) but significantly lower for HM3 (72%; HR, 10.82; 95% CI, 1.26‐92.68; *P* = .03). (Figure 2). However, survival with the HMII was comparable to the HM3 (83.7% vs. 88.4%; HR, 0.38; 95% CI, 0.076‐1.966; *P* = .25) and the HVAD (83.7% vs. 78.5%; HR, 0.73; 95% CI, 0.302‐1.782; *P* = .49) (Table 5).

## 4 DISCUSSION

DLI is still one of the most common complications during LVAD support,^1^, ^10^ and the risk of infection has been shown to increase steadily with longer periods of support.^11^, ^12^ The majority of studies evaluating risk factors for DLI have been conducted with pulsatile VADs^4^ and data for continuous flow devices are limited. Therefore, the aim of this study was to investigate the incidence of DLI‐related readmissions and to identify possible DLI risk factors in three contemporary continuous flow LVADs (HMII, HM3, and HVAD) as well as their effect on clinical outcomes.

DLI as a primary cause of readmission accounted for 12.6% at 2‐years following LVAD implantation (Figure 1). Similar to the results of Topkara et al,^7^ our data show that device‐related DLI was not an independent risk factor for survival in patients supported with HMII, HM3 or HVAD (Table 5). In contrast to previous studies in which higher age^13^ or BMI^14^, ^15^ was identified as a risk factor for DLI, our study did not show a significant association between DLI readmission and BMI or age—which is consistent with the results of Koval et al.^16^ However, older patients had a significantly higher risk (HR, 1.06; 95% CI, 1.006‐1.116; *P* = .03) for all‐cause death (Table 5). In addition, gender, diabetes, implant strategy, and INTERMACS level were not associated with DLI readmission. It is noteworthy that significantly more RDLI (65.2%) than patients without DLI (29.3%) had a smoking history, possibly because smokers may have poor wound healing, which makes them more susceptible to DLIs due to the lower oxygen content in subcutaneous wound tissue.^17^

In accordance with the results of Imamura et al,^18^ who identified serum albumin at discharge from the hospital as a predictor for DLI resumption, we found significantly lower albumin levels in RDLI (*P* < .001) compared to NRDLI and NoDLI patients (Table 1). Early detection and treatment of DLI can prevent sepsis and improve outcomes, so we analyzed laboratory parameters as potential predictors of DLI readmission up to 90 days before readmission. Compared to DLI readmission, leukocytes were significantly lower 90 days before (*P* = .019) but already at a comparable high level 60 days preceding readmission (*P* = .078). As shown in Table 4, fibrinogen in patients with DLI readmission 592 (220) mg/dL had a trend toward higher levels already 30 days before [500 (157) mg/dL] compared to 60 and 90 days before readmission, respectively [451 (224) and 496 (151) mg/dL]. Fibrinogen is a major risk factor for cardiovascular disease and is also an acute‐phase protein^19^ that can increase in response to infection and other stressful events.

These findings may be relevant to clinical practice since, as previously reported,^3^ the most common schedule for outpatient visits, especially in extra‐large VAD centers, is every 3 months or longer. Therefore, in patients with early signs of DLI and leukocytosis or elevated CRP (>1.3 mg/dL) and fibrinogen (>500 mg/dL) levels, proactive initiation of empirical antibiotic therapy may improve outcomes and prevent hospitalization even with pending exit culture. This is underlined by the fact that all NRDLI patients were successfully treated by non‐surgical strategies (66.7% antibiotics and 33.3% with silver dressing change) (Table 3).

The most common pathogens were Gram‐positive bacteria that colonize skin and adhere to implanted material and form a biofilm, especially *S. aureus* and *S. epidermis*, which cause >50% of DLI.^20^ This is consistent with our findings in RDLI (52%) and NRDLI patients (59.4%) with positive *S. aureus* microbiology. In accordance with Kusne et al,^4^ the most frequently reported Gram‐negative bacterium in our cohort was *P. aeruginosa* and occurred in 16% of RDLI and 9.4% of NRDLI patients.

When investigating risk factors for DLI readmissions, device type had a significant impact on the incidence of DLI readmission. Similar to the ENDURANCE trial^21^, we found no different hazard for DLI readmissions at 24 months between the HMII and HVAD (2% vs. 13%; HR, 4.52; 95% CI, 0.58‐35.02; *P* = .15) but significantly more DLI readmission in patients supported with the HM3 device (28%; HR, 10.82; 95% CI, 1.26‐92.68; *P* = .03) compared to the HMII. There are three possible reasons for this result: (a) driveline diameter size, (b) driveline material characteristics, and (c) overall geometry and connector setup, all influencing the mechanical stiffness of the percutaneous driveline. First, the HM3 has the largest driveline diameter of the outer (extracorporeal) pump cable (6.1 mm), compared to the HMII (5.7 mm) and HVAD (4.6 mm). Second, Imamura et al in^22^ showed that driveline stiffness could be an important factor in the context of DLI. The HMII driveline had only 20%‐25% of stiffness compared to other devices (EVAHEART and DuraHeart) and the highest driveline infection‐free rate among those three devices.^22^

In addition, the HMII driveline is only made of soft silicone, whereas the more rigid materials polyurethane + silicone used in the HM3 and the polyurethane material in the HVAD driveline potentially lead to more trauma and force applied to the driveline exit site during activities of daily living such as changing clothes, showering, changing bags, etc or unintentionally while sleeping or more generally through the behavior of each patient and the management of the VAD peripherals. Third, the rigid modular HM3 driveline connector might apply additional traction on the driveline exit site, which could lead to a higher risk of DLI in HM3 patients. In contrast to our study, in the MOMENTUM 3 final report^23^ no significant differences in DLI between HMII (19.4%) and HM3 (23.3%) were found. It is noteworthy that the HM3 DLI rates in the MOMENTUM 3 study were comparable to our HM3 cohort, but HMII patients in our single‐center experience experienced even fewer DLI‐related readmissions than HM3 patients. Therefore, new driveline exit site dressing methods, including additional binders or anchoring devices, may be required, especially in HM3 patients, to prevent DLI.

Despite the different relative risks for DLI readmission depending on the device, most likely due to device‐specific driveline characteristics, the device type was not a significant risk factor for 2‐year survival after HMII, HM3 or HVAD implantation (*P* = .26) (Table 5). Further investigations should be performed to investigate the influence of driveline materials and their mechanical properties as a risk factor for DLIs.

Our study has limitations, including its retrospective design, the limitation of data collection to first driveline infections and readmissions, as no recurrent events were analyzed. In addition, data analysis was limited to available variables in the medical records and analysis of patients from a single‐center, so the bias in patient selection may have influenced the outcomes after LVAD implantation. In particular, the small number of HM3 patients recently implanted may have been another factor of bias, and therefore a larger multicenter study should provide a more detailed description of risk factors for DLI readmission than was possible in this study.

## 5 CONCLUSIONS

DLI as the primary cause of readmission remains a serious problem following continuous‐flow LVAD implantation, but the occurrence of DLI had no effect on mortality. CRP, leukocytes, and fibrinogen were significantly higher in readmitted DLI patients and could be a risk factor as early as 60 days before readmission. HM3 patients had a higher risk for DLI‐related readmissions compared to HVAD or HMII, possibly due to device‐specific differences in the driveline features.

## CONFLICT OF INTEREST

TS, DW, and DZ are consultants for Medtronic Inc and Abbott Inc. None of the other authors has any financial relationship to disclose.

## AUTHOR CONTRIBUTIONS

All authors performed critical revision of the article and approved the final version.

*Concept and design:* Schlöglhofer, Zimpfer, Moscato

*Statistical analysis:* Schlöglhofer, Michalovics, Moscato

*Funding:* Zimpfer, Laufer, Schima, Moscato

*Drafted the article:* Schlöglhofer

*Collected the data:* Schlöglhofer, Michalovics, Riebandt, Angleitner, Stoiber, Wiedemann

## Supporting information

Supplementary Material

## References

[1] Kormos RL , Cowger J , Pagani FD , Teuteberg JJ , Goldstein DJ , Jacobs JP , et al. The Society of Thoracic Surgeons Intermacs database annual report: evolving indications, outcomes, and scientific partnerships. J Heart Lung Transplant. 2019;38:114–26. 30691593 10.1016/j.healun.2018.11.013

[2] Kirklin JK , Pagani FD , Kormos RL , Stevenson LW , Blume ED , Myers SL , et al. Eight annual INTERMACS report: special focus on framing the impact of adverse events. J Heart Lung Transplant. 2017;36:1080–6. 28942782 10.1016/j.healun.2017.07.005

[3] Schlöglhofer T , Robson D , Bancroft J , Sørensen G , Kaufmann F , Sweet L , et al. International coordinator survey results on the outpatient management of patients with the HeartWare^®^ ventricular assist system. Int J Artif Organs. 2016;39:553–7. 28058699 10.5301/ijao.5000538

[4] Kusne S , Mooney M , Danziger‐Isakov L , Kaan A , Lund LH , Lyster H , et al. An ISHLT consensus document for prevention and management strategies for mechanical circulatory support infection. J Heart Lung Transplant. 2017;36:1137–53. 28781010 10.1016/j.healun.2017.06.007

[5] Akhter SA , Badami A , Murray M , Kohmoto T , Lozonschi L , Osaki S , et al. Hospital readmissions after continuous‐flow left ventricular assist device implantation: incidence, causes, and cost analysis. Ann Thorac Surg. 2015;100:884–9. 26095106 10.1016/j.athoracsur.2015.03.010

[6] Hannan MM , Husain S , Mattner F , Danziger‐Isakov L , Drew RJ , Corey GR , et al. Working formulation for the standardization of definitions of infections in patients using ventricular assist devices. J Heart Lung Transplant. 2011;30:375–84. 21419995 10.1016/j.healun.2011.01.717

[7] Topkara VK , Kondareddy S , Malik F , Wang IW , Mann DL , Ewald GA , et al. Infectious complications in patients with left ventricular assist device: etiology and outcomes in the continuous‐flow era. Ann Thorac Surg. 2010;90:1270–7. 20868826 10.1016/j.athoracsur.2010.04.093

[8] Toda K , Sawa Y . Clinical management for complications related to implantable LVAD use. Gen Thorac Cardiovasc Surg. 2015;63:1–7. 25370679 10.1007/s11748-014-0480-0

[9] Bernhardt AM , Schlöglhofer T , Lauenroth V , Mueller F , Mueller M , Schoede A , et al. Prevention and early treatment of driveline infections in ventricular assist device patients—the DESTINE staging proposal and the first standard of care protocol. J Crit Care. 2020;56:106–12. 31896443 10.1016/j.jcrc.2019.12.014

[10] Kusne S , Staley L , Arabia F . Prevention and infection management in mechanical circulatory support device recipients. Clin Infect Dis. 2017;64:222–8. 27986679 10.1093/cid/ciw698

[11] Zierer A , Melby SJ , Voeller RK , Guthrie TJ , Ewald GA , Shelton K , et al. Late‐onset driveline infections: the Achilles’ heel of prolonged left ventricular assist device support. Ann Thorac Surg. 2007;84:515–20. 17643627 10.1016/j.athoracsur.2007.03.085

[12] Sharma V , Deo SV , Stulak JM , Durham LA , Daly RC , Park SJ , et al. Driveline infections in left ventricular assist devices: implications for destination therapy. Ann Thorac Surg. 2012;94:1381–6. 22818961 10.1016/j.athoracsur.2012.05.074

[13] Schaffer JM , Allen JG , Weiss ES , Arnaoutakis GJ , Patel ND , Russell SD , et al. Infectious complications after pulsatile‐flow and continuous‐flow left ventricular assist device implantation. J Heart Lung Transplant. 2011;30:164–74. 20888258 10.1016/j.healun.2010.08.003

[14] John R , Aaronson KD , Pae WE , Acker MA , Hathaway DR , Najarian KB , et al. Drive‐line infections and sepsis in patients receiving the HVAD system as a left ventricular assist device. J Heart Lung Transplant. 2014;33:1066–73. 25087103 10.1016/j.healun.2014.05.010

[15] Raymond AL , Kfoury AG , Bishop CJ , Davis ES , Goebel KM , Stoker S , et al. Obesity and left ventricular assist device driveline exit site infection. ASAIO J. 2010;56:57–60. 20051832 10.1097/MAT.0b013e3181c879b1

[16] Koval CE , Thuita L , Moazami N , Blackstone E . Evolution and impact of drive‐line infection in a large cohort of continuous‐flow ventricular assist device recipients. J Heart Lung Transplant. 2014;33:1164–72. 25034793 10.1016/j.healun.2014.05.011

[17] Sorensen LT , Karlsmark T , Gottrup F . Abstinence from smoking reduces incisional wound infection: a randomized controlled trial. Ann Surg. 2003;238:1–5. 12832959 10.1097/01.SLA.0000074980.39700.31 PMC1422652

[18] Imamura T , Kinugawa K , Nitta D , Inaba T , Maki H , Hatano M , et al. Readmission due to driveline infection can be predicted by new score by using serum albumin and body mass index during long‐term left ventricular assist device support. J Artif Organs. 2015;18:120–7. 25604148 10.1007/s10047-015-0816-2

[19] Baumann H , Gauldie J . The acute phase response. Immunol Today. 1994;15:74–80. 7512342 10.1016/0167-5699(94)90137-6

[20] Toba FA , Akashi H , Arrecubieta C , Lowy FD . Role of biofilm in *Staphylococcus aureus* and *Staphylococcus epidermidis* ventricular assist device driveline infections. J Thorac Cardiovasc Surg. 2011;141:1259–64. 20709333 10.1016/j.jtcvs.2010.07.016 PMC2988078

[21] Rogers JG , Pagani FD , Tatooles AJ , Bhat G , Slaughter MS , Birks EJ , et al. Intrapericardial left ventricular assist device for advanced heart failure. N Engl J Med. 2017;376:451–60. 28146651 10.1056/NEJMoa1602954

[22] Imamura T , Murasawa T , Kawasaki H , Kashiwa K , Kinoshita O , Nawata K , et al. Correlation between driveline features and driveline infection in left ventricular assist device selection. J Artif Organs. 2017;20:34–41. 27448017 10.1007/s10047-016-0923-8

[23] Mehra MR , Uriel N , Naka Y , Cleveland JC , Yuzefpolskaya M , Salerno CT , et al. A fully magnetically levitated left ventricular assist device—final report. N Engl J Med. 2019;380:1618–27. 30883052 10.1056/NEJMoa1900486

