# Supplementary material for: Left ventricular assist device driveline infections in three contemporary devices
Source: Artif Organs. 2020 Nov 28;45(5):464–72. doi: 10.1111/aor.13843 (PMC8247301; doi:10.1111/aor.13843)
Supplement: Supplementary file 1 — Supplementary Material [file AOR-45-464-s001.docx]

**Supplementary Data S1:** Microbiological profiles of patients with driveline infection stratified by readmission due to infection

| **Pathogen Type** | **DLI Readmission (n=25 pathogens)** | **DLI**  **No Readmission (n=32 pathogens)** |
| --- | --- | --- |
| *Staphylococcus aureus* | 13 (52.0) | 19 (59.4) |
| *Pseudomonas aeruginosa* | 4 (16.0) | 3 (9.4) |
| *Proteus mirabilis* | 2 (8.0) | 1 (3.1) |
| *Staphylococcus epidermis* | 1 (4.0) | 4 (12.5) |
| *Bacteroides vulgatus* | 1 (4.0) | - |
| *Corynebacterium amycolatum* | 1 (4.0) | 1 (3.1) |
| *Corynebacterium* species | 1 (4.0) | 1 (3.1) |
| *Enterococcus faecalis* | 1 (4.0) | - |
| *Fusobacterium nucleatum* | 1 (4.0) | - |
| *Enterobacter cloacae* | - | 2 (6.3) |
| *Staphylococcus* coagulase negative | - | 1 (3.1) |

Data presented as n (%). DLI readmission: 25 pathogens detected in 23 patients. DLI no readmission: 32 pathogens detected in 27 patients.

DLI, Pump-related percutaneous driveline infection
